# Omalizumab for successful chemotherapy desensitisation: What we know so far

**DOI:** 10.1002/clt2.12086

**Published:** 2021-12-13

**Authors:** Roxana Silvia Bumbacea, Selda Ali, Sabina Loredana Corcea, Luiza Spiru, Cornelia Nitipir, Victor Strambu, Dragos Bumbacea

**Affiliations:** ^1^ “Carol Davila” University of Medicine and Pharmacy Bucharest Romania; ^2^ Department of Allergy and Clinical Immunology “Dr. Carol Davila” Nephrology Clinical Hospital Bucharest Romania; ^3^ Novomedica Center of Excellence Bucharest Romania; ^4^ The Excellence Memory Center and Longevity Medicine “Ana Aslan” International Foundation Bucharest Romania; ^5^ Department of Oncology Elias University Emergency Hospital Bucharest Romania; ^6^ Department of General Surgery “Dr. Carol Davila” Nephrology Clinical Hospital Bucharest Romania; ^7^ Department of Pneumology and Acute Respiratory Care Elias Emergency University Hospital Bucharest Romania

**Keywords:** chemotherapy hypersensitivity, drug desensitisation, omalizumab, désensibilisation aux médicaments, hypersensibilité à la chimiothérapie, omalizumab

## Abstract

**Background:**

Hypersensitivity reactions induced by chemotherapeutic drugs may influence the course of the oncologic disease by preventing doctors from prescribing first‐line therapy. In order to prevent another hypersensitivity reaction to the culprit chemotherapeutic agent, the physician can decide between two possibilities: premedication or desensitisation protocols. Rapid drug desensitisation showed successful results for most patients, but some of them may develop symptoms. Although omalizumab is not licensed as premedication or adjuvant therapy in chemotherapy desensitisation protocols, there have been published some case reports and small sample size studies that indicated promising results.

**Methods:**

We reviewed all the published literature regarding the use of omalizumab during chemotherapy desensitisation protocols.

**Results and conclusions:**

We found a great heterogeneity between the doses and the interval between omalizumab injections and chemotherapy ‐ rapid drug desensitisation, but most of the studies showed promising results. As a corollary, we propose a dose regimen of omalizumab administered before the first desensitisation protocol. Then, omalizumab should be administered one day before every chemotherapy regimen. Omalizumab might be used as an adjuvant therapy and might be a solution for a hopeless situation.

## 1 INTRODUCTION

Chemotherapy (CHT) is one of the basic medical approaches for treating patients with different types of cancer. Chemotherapy agents are used either alone or in combination with targeted therapies constituted by monoclonal antibodies or other biologicals.^1^

Hypersensitivity reactions (HSRs) induced by CHT can be defined as unpredicted signs and symptoms not consistent with a toxicity reaction.^2^ The mechanisms responsible for HSRs are not fully understood and may vary between IgE‐mediated, non‐IgE‐mediated or unclear pathogenic events.^3^

Almost all CHT drugs can induce HSRs, but they are reported only in about 5% of patients; this percentage may be substantially underestimated because of unreported mild and moderate reactions.^4^ According to recent data, CHT‐induced HSRs are the third leading cause of fatal drug‐induced anaphylaxis in the United States^5^; in Europe they are amongst top 5 inducers.^6^

Based on the risk of generating HSRs, it is possible to divide the chemotherapeutic agents into three groups: drugs with high, intermediate, or low potential to cause HSRs, as summarized in Table 1. The reactions can be caused by the parent compound, its metabolites, or by the solvent. Depending on this grouping, the problem of CHT‐induced HSRs is notable for patients treated with platinum compounds, taxanes, L‐asparaginase, epipodophyllotoxins and is lower for others.^7^

**TABLE 1:** Classificasion of chemotherapeutic agents based on risk to induce hypersensitivity reactions^7^

Drugs with high potential	Drugs with intermediate potential	Drugs with low potential
Platinum compounds	Anthracyclines	Cytarabine
Oxaliplatin	Doxorubicin	Cyclophosphamide
Carboplatin	Daunorubicin	Ifosfamide
Cisplatin	Epirubicin	
Taxanes	Idarubicin	
Paclitaxel	6‐Mercaptopurine	
Docetaxel	Azathioprine	
Other cytostatic drugs	Methotrexate	
L‐Asparaginase		
Procarbazine		
Epipodophyllotoxins		
Teniposide		
Etoposide		

Unlike other drugs that can be easily replaced when a HSR occurs, CHT drugs are often unique and essential in the treatment of neoplastic disease and thereby the management of HSRs to CHT is crucial.^8^

The diagnosis of HSR is based on history, in vivo tests (skin prick, intradermal and provocation tests) and in vitro tests. The clinical manifestations are variable, unpredictable and involve the skin (e.g., rash, pruritus, urticaria, angioedema, palmar erythema, facial flushing), the respiratory tract (e.g., bronchospasm), the gastrointestinal tract (e.g., abdominal pain, nausea, diarrhea), and the cardiovascular system (alterations in blood pressure and heart rate).^9^ Severe reactions usually develop during the infusion of the chemotherapy and most of them are IgE‐mediated as it has been clearly demonstrated for platinum salts,^10^, ^11^ whereas mild to moderate reactions can occur either during treatment or in the 24 up to 72 h after CHT administration and are apparently related to other mechanisms like mast cell and basophil activation and degranulation or complement activation.^12^, ^13^

In respect of skin tests usefulness, their importance has been proved in platin salts‐induced HSRs, demonstrating an IgE‐mediated mechanism particularly for carboplatin and oxaliplatin.^14^ Prick and intradermal skin tests to platinum drugs are valuable diagnostic tools; their high sensitivity has been shown by several studies on carboplatin (66%–100%) and oxaliplatin (26%–100%).^15^, ^16^ In addition, the severity of the reaction appears to be related to the sensitivity of skin tests.^11^

In clinical practice, if the first‐line treatment led to a HSR, the oncologist might switch to a second line therapy which can be less effective and can lead to significant morbidity.^17^ However, if the culprit drug is associated with increased life expectancy and increased quality of life or if there is no therapeutic alternative, the physician must weigh the benefit of continuing the treatment against the risk of a potential fatal anaphylactic reaction during the following administration of chemotherapy.^7^

In order to prevent another HSR to the culprit chemotherapeutic agent, the physician can decide between two possibilities: premedication or desensitisation protocols.

## 2 PREMEDICATION

Premedication schedules are carried out to prevent HSRs; they include administration of corticosteroids and antihistamines prior to chemotherapy infusion. Premedication is effective as well as recommended to prevent HSRs to different CHT, such as taxanes, epipodophillotoxins and pegasparaginase.^7^, ^18^ This procedure has dramatically decreased the incidence of HSRs to taxanes down to 2%–4% of cases.^19^ Instead, for platinum salts, premedication is ineffective in preventing IgE‐mediated HSRs.^20^, ^21^

Occasionally, in case of premedication failure or if the procedure cannot be implemented, a drug desensitisation protocol could be recommended. Rapid drug desensitisation (RDD) is the best option for mast cell‐mediated HSRs, whether the involved mechanism is IgE‐mediated or not.^17^

## 3 DESENSITISATION TO CHEMOTHERAPEUTIC AGENTS

RDD is a procedure that induces a temporary tolerance to a drug, by consecutive administration of small doses of the culprit drug until the total therapeutic dose is reached.^15^

RDD induces transient unresponsiveness, so patients need to be re‐desensitised each time they are re‐exposed to the culprit CHT. Throughout RDD, patients with IgE and non‐IgE HSRs can safely receive needed medication while minimizing or completely inhibiting adverse reactions. Due to the extensive clinical usefulness and success of RDD, the molecular mechanisms of inducing temporary tolerance have been extensively investigated but are still incompletely understood.^17^

The standardized 12‐step protocol is the most used in RDD and can help achieve a temporary tolerance of the drug; the protocol usually starts with 1/1000 from the final dose and subsequently, the doses are doubled at each step at fixed time intervals.^17^, ^22^

Patients who have had severe anaphylactic reactions to CHT or who have reacted at an early stage in the standard 12‐step RDD, may experience fewer symptoms when using a 16‐step protocol according to Mariana Castells' one.^17^

RDD showed successful results for most patients, but some of them may develop symptoms despite intense pre‐treatment and extra antiallergic medication received during the desensitisation procedure. For these special cases, omalizumab might be used as an adjuvant therapy to induce CHT tolerance.

Omalizumab has also been used in achieving tolerance to other drugs such as insulin, elosulfase ɑ, acetylsalicylic acid.^23^, ^24^, ^25^ The largest evidence is regarding the use of omalizumab in acetylsalicylic acid desensitisation for aspirin exacerbated respiratory disease.^25^

## 4 OMALIZUMAB

Omalizumab is a recombinant humanized anti‐IgE monoclonal antibody that specifically binds to the C‐ε‐3 domain of free IgE and the surface IgE of IgE expressing B cells but not to IgE bound to high or low affinity IgE receptors (FcεRI, respectively FcεRII) and therefore they do not trigger effector cell degranulation.^26^ The complexes formed between omalizumab and IgE result in a significant decrease in free IgE in serum. They also prevent IgE from binding to effector cells, resulting in decreased mediator release in response to allergens^27^ and in a reversible downregulation of FcεRI receptors on basophils, mast cells, and dendritic cells.^28^, ^29^ Recent studies have shown that Omalizumab is able to detach IgE from high affinity IgE receptors, although the exact mechanism is still under investigation.^30^

It was approved for the treatment of severe allergic asthma, chronic spontaneous urticaria and severe chronic rhinosinusitis with nasal polyps. In addition, it has been studied as an add‐on therapy for immunotherapy and other disorders such as food allergy, atopic dermatitis, idiopathic anaphylaxis, inducible urticaria, allergic bronchopulmonary aspergillosis and mastocytosis.^31^, ^32^ The studies' results suggest that anti‐IgE monoclonal antibodies may have important immunotherapeutic benefits for the management of the above‐mentioned disorders. Although the complete mechanism of action of omalizumab is not fully understood, some authors suggest that it may reduce mast cell releasability, it reverses basopenia and improves basophil IgE receptor function. Also, it may reduce the activity of IgG autoantibodies against FcεRI and IgE.^33^

## 5 OMALIZUMAB IN CHEMOTHERAPY‐RDD: CLINICAL EVIDENCE

Although omalizumab is not licensed as premedication or adjuvant therapy in CHT desensitisation protocol, there have been published some case reports and small sample size studies that indicated promising results. We have reviewed articles, published abstracts, and conference proceedings on Pubmed, Embase, Cochrane Library and Web of Science that were published before June 10, 2021. Medical Subject Heading and keywords were used together, including omalizumab, chemotherapy, oxaliplatin, carboplatin, desensitisation. This approach was also combined with a manual search of references in all selected studies. Until now, there have been published two clinical trials and ten case reports.^34^, ^35^, ^36^, ^37^, ^38^, ^39^, ^40^, ^41^, ^42^, ^43^, ^44^, ^45^ All the results are synthesized chronologically in Table 2.

**TABLE 2:** Omalizumab in chemotherapy desensitisation—current data

Author (Ref.)	Patients	Drug involved	Omalizumab dose	Interval between omalizumab doses (days)	Interval between omalizumab and CHT (days)	Number of omalizumab doses	Number of desensitisation protocols	Tolerance
Cahill, 2012^34^	1	Oxaliplatin	150 mg	14	UNK	6	5	Mild reactions
Ojaimi, 2014^35^	1	Carboplatin	300 mg	14	1	9	5	Yes
Saura, 2016^36^	3	Carboplatin	UNK	UNK	UNK	UNK	15	Yes
Garcia, 2016^37^	1	Carboplatin	UNK	UNK	UNK	UNK	UNK	Mild reaction
Hong, 2018^38^	5	CHT (platins, taxanes) or monoclonal antibodies	300 mg	28	UNK	3	3	Mild reactions
Stein, 2018^39^	9	Oxaliplatin	300 mg	14	7	Median = 5 (1–10)	UK	Yes
Prieto‐Garcia, 2019^40^	1	Oxaliplatin	300 mg	15	7	13	13	Yes
De Las Vaceillas Sánchez, 2019^41^	1	Oxaliplatin	600/300 mg	14	1	6	5	Yes
Cuevas, 2019^42^	1	Oxaliplatin	300 mg	UNK	6	1	1	Anaphylaxis
Sanchez‐Morillas, 2020^43^	2	Carboplatin	300/150 mg	7/14^a^	1	9	6	Yes
		Carboplatin	300/150 mg	7/14^a^	1	6	4	Mild reaction
Oude Elberink, 2020^44^	1	Carboplatin	300 mg	14	11^b^	9	9	Yes
Penella, 2020^45^	1	Oxaliplatin	300 mg	14	19/14^c^	5	5	Yes

Abbreviations: CHT, chemotherapy; RDD, rapid drug desensitisation; UNK, unknown.

^a^ 7 days between 300 and 150 mg of Oma; 14 days between the next 150 mg doses.

^b^ Before first desensitisation.

^c^ 19 days between the first dose of Oma and CHT‐RDD, then 14 days.

The first description of omalizumab use as premedication for oxaliplatin desensitisation was made by Cahill et al.^34^ in a 68‐year‐old patient with colon cancer who developed anaphylaxis to oxaliplatin. Initially, the patient received two doses of 150 mg omalizumab at 2 weeks interval, before a 16‐step desensitisation protocol. He tolerated four subsequent oxaliplatin desensitisation protocols with only mild reactions.

Ojaimi et al.^35^ reported the case of a 63‐year‐old non‐atopic female who failed different desensitisation protocols to carboplatin, but after receiving premedication with omalizumab, she successfully completed five RDD (modified over 4 days). The authors used a series of nine fortnightly doses of 300 mg of omalizumab given subcutaneously. The first RDD was administered 1 day after the third dose of omalizumab. This is the only report that monitored both skin tests to carboplatin and total IgE values. Intradermic skin tests to carboplatin 10 mg/ml became negative and the value of total IgE decreased at the end of the study.

A series of three patients who benefited from pre‐treatment with omalizumab before chemotherapy desensitisation was reported by Saura et al.^36^ All three cases had a history of carboplatin anaphylactic reaction (proven by tryptase elevation) during classical desensitisation protocols. By using omalizumab before RDD, all three patients tolerated the total number of 15 desensitisation procedures.

Another case report of successful use of omalizumab as premedication to carboplatin desensitisation was described by Garcia et al in a 52‐year‐old female.^37^

Hong's study^38^ included five patients who had IgE‐mediated reaction to CHT including platins, taxanes, or monoclonal antibodies and a breakthrough reaction during desensitisation protocol. The participants received a dose of 300 mg omalizumab every four weeks, at three consecutive visits. There is no information about the time frame between omalizumab administration and CHT‐RDD, except that the two drugs were not given on the same day. Out of these five patients, only three concluded the study protocol (one dropped out due to cytokine storm‐like reaction and another due to disease progression). The investigator concluded that omalizumab did not completely abrogate breakthrough reactions in all patients, but the symptoms were milder and the three patients reported significantly less symptoms during desensitisation protocols.

Stein et al.^39^ reported a small sample size study that enrolled nine patients diagnosed with stage IV colon cancer and oxaliplatin HSRs during chemotherapy protocol. Omalizumab was administered in a fixed dose of 300 mg every 2 weeks, alternating with Oxaliplatin chemotherapy, administered also at 2 weeks interval. First dose of omalizumab was administered 7 days prior to Oxaliplatin. Only one patient experienced a HSR during the first cycle of oxaliplatin. The other patients were able to tolerate the desensitisation protocols, the median number of cycles was 5 (range 1–10). The reasons for dropping out were disease progression or chemotherapy related side effects.

Omalizumab was also helpful in achieving tolerance to oxaliplatin in a patient who suffered anaphylaxis after completing the desensitisation protocol. Omalizumab 300 mg was administered every 15 days, one week before the chemotherapeutic agent and the patient was able to tolerate 13 more cycles.^40^

A different dose regimen was used by De Las Vecillas Sánchez et al.^41^ in a patient with severe anaphylaxis (with elevated tryptase level) during desensitisation protocol to oxaliplatin. Two doses of omalizumab (600 mg and after 2 weeks another 300 mg) were administered 15 days, respectively 1 day prior to RDD. The patient had a mild reaction with slight increase of the tryptase level; subsequently, she tolerated four more RDD with omalizumab premedication with no noticeable reactions. The high sensitisation to oxaliplatin and the elevated total IgE (>5000 kU/L) might be the reason for deciding on the high initial dose of omalizumab.

The only unsuccessful reported omalizumab‐RDD was described in a patient who received 300 mg 6 days prior to oxaliplatin desensitisation.^42^ Clinical symptoms and increased tryptase levels were reported during the first step of the desensitisation protocol. No other attempt to achieve tolerance was noted.

Sanchez‐Morillas et al.^43^ reported two patients with severe anaphylactic reactions during CHT (carboplatin) who were premedicated with omalizumab in RDD. The authors did not recommend a previous RDD in the absence of omalizumab premedication. Initially, both patients received 300 mg omalizumab; 7 days later they received another 150 mg. This course of action was performed twice a month, until the end of chemotherapy.

The 16 steps chemotherapy desensitisation protocols were performed 24 h after the second dose of omalizumab. The first patient tolerated six cycles of carboplatin desensitisation, while the second one was able to finish only four cycles but with mild cutaneous reactions that were resolved with antihistamines and corticosteroids. As a conclusion, for patients with a history of CHT‐induced severe anaphylactic reaction, the authors propose a bimonthly 300 mg omalizumab premedication protocol, with at least one dose administered before the first RDD.^43^

This premedication plan was adapted by Oude Elberink et al.^44^ in a patient with a successful carboplatin desensitisation schedule after receiving omalizumab 300 mg fortnightly, with the first dose given 11 days before the RDD. Interestingly, in this case report, omalizumab was also used as premedication when a new cycle of desensitisation protocol to carboplatin was needed because of the relapse of the ovarian cancer.

Prolonging the desensitisation protocol to oxaliplatin alongside premedication with 300 mg omalizumab have proven successful in a patient with metastatic sigmoid adenocarcinoma who experienced anaphylaxis during standard desensitisation. On the first cycle, the time frame between omalizumab administration and chemotherapy was 19 days, the interval being shortened to 14 days on four subsequent administrations. The authors highlighted the decrease of total IgE value from 4690 to 575 kU/L.^45^

## 6 DISCUSSION

CHT‐RDD must be used for oncologic patients when no other therapeutical options with the same benefits are available. In case of RDD failure, omalizumab might be used as an adjuvant therapy and might be a solution for a hopeless situation. Although the main anti‐IgE mechanism of action of omalizumab is well defined, its complete mastermind of processes in other diseases, including in RDD, is still enigmatic.

It makes sense to try inducing tolerance by using omalizumab in patients who fail platinum salts desensitisation, because the underlying mechanism of platinum salts‐induced hypersensitivity is IgE‐mediated. All the aforementioned studies included patients diagnosed with platinum salt‐hypersensitivity. The selection of omalizumab dose was independent of the patients' weight or the total IgE values, opposed to asthma or nasal polyposis. Instead, the choice of omalizumab dose was like the one used in chronic urticaria.

We found a great heterogeneity between the doses and the interval between omalizumab injections and CHT‐RDD; some studies used the anti‐IgE therapy only before the first RDD, but most of the studies used it before all the subsequent RDD.

Most of the authors preferred to administer omalizumab fortnightly, except Hong et al.^38^ The time interval between the last omalizumab injection and the desensitisation protocol varied largely, ranging from 24 h to 19 days. Some of the authors preferred using every other week regimen for omalizumab, while administering the chemotherapy as prescribed by the oncologist every 21 days.^35^, ^44^

**FIGURE 1:**
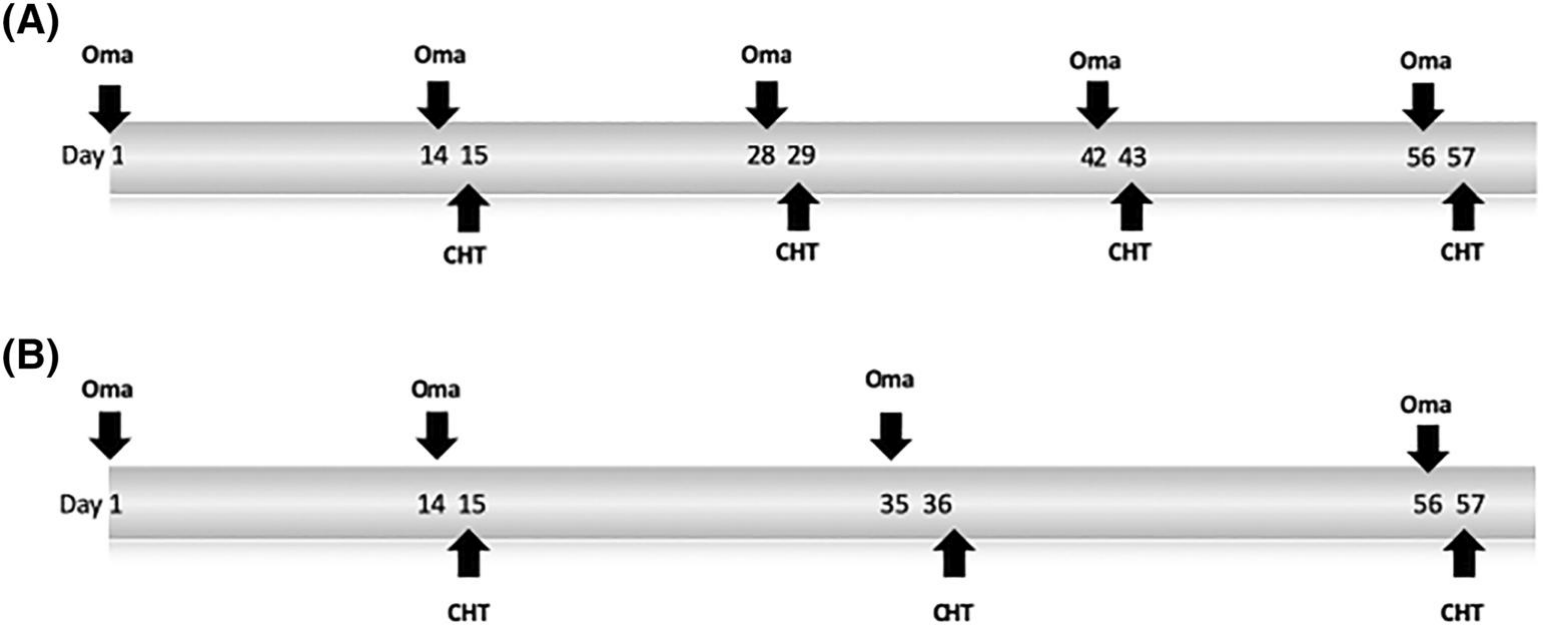
Timeframe between Omalizumab (Oma) and CHT‐RDD: (A) Patients whose CHT regimen is every 14 days and (B) Patients whose CHT regimen is every 21 days. CHT, chemotherapy; RDD, rapid drug desensitisation

As a corollary of these results, we propose a dose regimen of 300 mg omalizumab administered twice before the first CHT‐RDD, 15 days and 1 day prior to the first CHT infusion. Then, omalizumab should be administered one day before the CHT regimen, while the interval between CHT might vary depending on the drug used in the respective oncologic disease (Figure 1).

The results of most of the studies were promising, the patients tolerated subsequent CHT‐RDD without HSRs or accompanied by mild reactions only. Unfortunately, a homogeneous definition of mild reactions was not given in the above‐mentioned studies.

## 7 FINAL REMARKS

RDD has become a key component of the management of CHT‐HSR. It is the only effective approach for overcoming the HSR to first‐line therapy, thus representing an important progression in patients' treatment and prognosis. Understanding the mechanism of action implied to RDD will allow improvement in patients' treatment.

Omalizumab, an anti‐IgE drug, may be used as an adjuvant therapy in RDD for patients with IgE‐mediated CHT‐HSRs in troublesome desensitisations despite premedication. Although the above‐mentioned case reports and small size clinical trials showed promising results, further studies with larger number of patients are necessary for setting up standard recommendations for omalizumab adjuvant‐RDD protocol.

## CONFLICT OF INTEREST

All authors declare no conflict of interest.

## AUTHOR CONTRIBUTIONS

Roxana Silvia Bumbacea and Dragos Bumbacea designed and supervised the study. Selda Ali, Sabina Loredana Corcea and Roxana Silvia Bumbacea performed the research. Selda Ali, Sabina Loredana Corcea, Cornelia Nitipir and Victor Strambu reviewed the references. Selda Ali, Sabina Loredana Corcea, Victor Strambu and Luiza Spiru wrote the manuscript. Luiza Spiru, Sabina Loredana Corcea and Victor Strambu contributed to tables and figure. Dragos Bumbacea and Roxana Silvia Bumbacea revised the article critically for important intellectual content. All authors read and approved the final manuscript for publication

## Data Availability

The data that support the findings of this study are available from the corresponding author upon reasonable request.
